# Glycosylation deficiency of lipopolysaccharide-binding protein and corticosteroid-binding globulin associated with activity and response to treatment for rheumatoid arthritis

**DOI:** 10.1186/s12967-019-02188-9

**Published:** 2020-01-06

**Authors:** Federica Ciregia, Dominique Baiwir, Gaël Cobraiville, Thibaut Dewael, Gabriel Mazzucchelli, Valérie Badot, Silvana Di Romana, Paschalis Sidiras, Tatiana Sokolova, Patrick Durez, Michel G. Malaise, Dominique de Seny

**Affiliations:** 1 Laboratory of Rheumatology, GIGA-I3, University of Liège, CHU de Liège, 4000 Liège, Belgium; 2 GIGA Proteomic Facility, University of Liège, 4000 Liège, Belgium; 3 Mass Spectrometry Laboratory, System Biology and Chemical Biology, GIGA-Research, University of Liège, 4000 Liège, Belgium; 4 Department of Rheumatology, CHU Brugmann, 1200 Brussels, Belgium; 5 Department of Rheumatology, CHU Saint-Pierre, 1200 Brussels, Belgium; 6 Department of Rheumatology, Hôpital Erasme, Université Libre de Bruxelles, 1200 Brussels, Belgium; 7 Department of Rheumatology, Cliniques Universitaires Saint-Luc, Institut de Recherche Expérimentale et Clinique (IREC), Université Catholique de Louvain, 1200 Brussels, Belgium

**Keywords:** Rheumatoid arthritis, Post translational modifications, Glycosylation, CBG, LBP, Biomarkers

## Abstract

**Background:**

Serum protein glycosylation is an area of investigation in inflammatory arthritic disorders such as rheumatoid arthritis (RA). Indeed, some studies highlighted abnormalities of protein glycosylation in RA. Considering the numerous types of enzymes, monosaccharides and glycosidic linkages, glycosylation is one of the most complex post translational modifications. By this work, we started with a preliminary screening of glycoproteins in serum from RA patients and controls.

**Methods:**

In order to isolate glycoproteins from serum, lectin wheat germ agglutinin was used and quantitative differences between patients and controls were investigated by LC–MS/MS. Consequently, we focused our attention on two glycoproteins found in this explorative phase: corticosteroid-binding globulin (CBG) and lipopolysaccharide-binding protein (LBP). The subsequent validation with immunoassays was widened to a larger number of early RA (ERA) patients (n = 90) and well-matched healthy controls (n = 90).

**Results:**

We observed a significant reduction of CBG and LBP glycosylation in ERA patients compared with healthy controls. Further, after 12 months of treatment, glycosylated CBG and LBP levels increased both to values comparable to those of controls. In addition, these changes were correlated with clinical parameters.

**Conclusions:**

This study enables to observe that glycosylation changes of CBG and LBP are related to RA disease activity and its response to treatment.

## Background

Rheumatoid arthritis (RA) is a chronic inflammatory disease of unknown etiology characterized by a symmetrical joint synovitis resulting in joints swelling, pain, disability and progressive joint damage [1]. Given the presence of autoantibodies (e.g. rheumatoid factor, anti-citrullinated protein antibody), RA is considered as an autoimmune disease [2]. The articular inflammatory load and autoimmunity bring to the destructive progression of the disease. The latest American and European College of Rheumatology (ACR/EULAR) criteria for classification focused on features at earlier stages of disease considering the importance for earlier diagnosis and the introduction of effective disease-suppressing therapy such as the disease modifying antirheumatic drugs (DMARDs) [2]. Since the eighties, research on serum protein glycosylation has been an area of investigation in inflammatory arthritis diseases. Indeed, removal of terminal sugar residues (sialic acid and galactose) of human IgG glycosylated chains has been extensively studied and described as playing a pathogenic role in RA [3, 4]. Moreover, these changes followed the disease remission during pregnancy and treatment [5, 6].

Protein glycosylation, namely the covalent attachment of carbohydrate residue, is one of the most common post translational modification which influences structure, stability and function of proteins. Glycosylation is not under genetic control and its level reflects glycosidases and glycosyltransferases activity. The non-enzymatic glycosylation, referred as glycation, can also occur and is related to pathophysiological conditions [7–9]. It has been estimated that at least 16 enzymes, 13 different monosaccharides and 8 amino acid types participate in the formation of approximately 41 diverse glycosidic linkages, contributing to make glycosylation one of the most elaborate post translational modifications of proteins [10, 11]. Therefore, there are different subtypes of glycosylation, for which *N*- and *O*-linked glycosylation are the most characterized. *N*-Glycosylation is the specific bond of glycans to the amide residue of an asparagine in Asn-X-Ser/Thr sequence (X amino acid cannot be proline). *O*-Glycosylation is the specific and covalent bond of glycans onto oxygen of a Ser or a Thr residue [12, 13]. *N*- and *O*-linked glycosylation are finely regulated by transcriptional control of glycosyltransferases and glycosidases and their accessibility to substrates but also by the spatial compartmentalization of components related to glycosylation machinery [13, 14]. Aberrant glycosylation can occur in pathological conditions such as cancers [15, 16] and inflammation [11, 17–19]. Heretofore, some studies highlighted abnormalities of protein glycosylation in RA [20–22] and, recently, the shift in the glycosylation profile of serum transferrin in RA has been proposed as a biochemical marker of RA activity [23]. Therefore, with this work, we started with a preliminary screening of glycoproteins in serum from RA patients and healthy controls with a proteomics approach, followed by validation on early RA (ERA). Indeed, the current model for the treatment of the disease is to manage patients in the early stages of RA. Undoubtedly, patients benefit from an early diagnosis and treatment of RA which can decrease the progress of joint damage up to 90% [24]. We focused our attention on two glycoproteins found in the explorative phase: corticosteroid-binding globulin (CBG) and lipopolysaccharide (LPS)-binding protein (LBP). CBG, also known as transcortin or serpin 6, is the major carrier for glucocorticoids, binding up to 80% of circulating cortisol [25]. CBG has a key role in targeted cortisol delivery in RA and its steroid-binding activity is influenced by glycosylation [26, 27]. LBP is an acute phase protein which takes part to the innate immune response. Since 1995, LBP has been suggested as a new marker of synovial inflammation, playing a major role in joint disorders, and its levels in serum were found significantly higher in patients with RA [28, 29]. Moreover, recently, LBP has been proposed as a sensitive serum biomarker to evaluate RA disease activity [30]. Hence, the aim of the present study has been to assess the glycosylation changes of CBG and LBP in a larger cohort of ERA patients, and to evaluate their association with clinical characteristics and with the response to a treatment of 12 months. The goal was to succeed in verifying if glycosylation changes can reflect the course of RA.

## Methods

### Experimental design

**Fig. 1:**
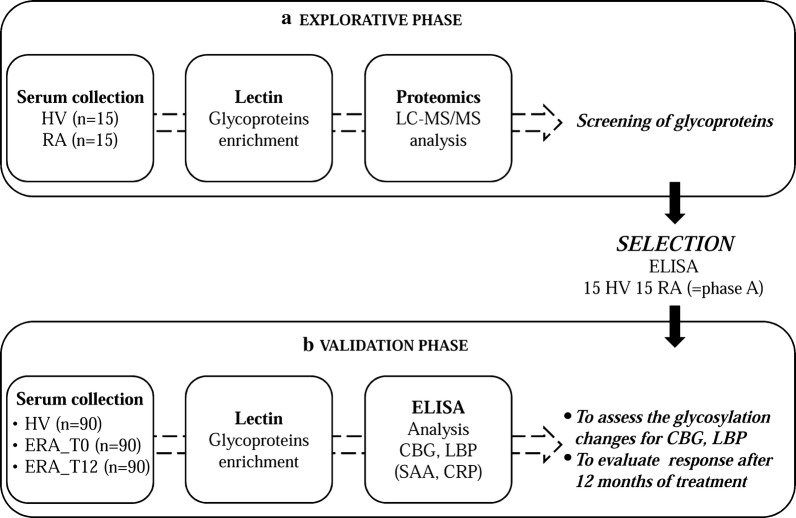
The graphical representation of the experimental design. *HV* healthy volunteers, *ERA_T0* early rheumatoid arthritis patients at time 0, *ERA_T12* the same ERA patients after 12 months of treatment, *CBG* corticosteroid-binding globulin, *LBP* lipopolysaccharide-binding protein, *SAA* serum amyloid A, *CRP* C-Reactive Protein

This glycoproteomics study can be divided in two main consecutive phases (i) the explorative phase and (ii) the validation phase (Fig. 1). The explorative phase was aimed to highlight glycosylation changes in RA respect to healthy volunteers (HV). For this purpose, glycoproteins were isolated from serum samples (15 RA, 15 HV) by using wheat germ agglutinin (WGA), a lectin with high affinity for sialic acid, and analysed by a proteomics approach, LC–MS/MS. The statistical analysis of proteomics data and ELISA assays let us to focus on two glycoproteins whose sialylation level was modified in RA respect to HV: CBG and LBP. Afterwards, in the validation phase, ELISA assays were carried out to assess if these glycosylation changes could be confirmed in a bigger cohort of subjects (90 RA, 90 HV) and to evaluate the status of glycosylation of both proteins after 1 year of treatments.

### Patients

#### Glycoproteins selection

With the purpose of the glycoproteins selection, 15 women were consecutively recruited through hospital outpatient clinics. All RA patients fulfilled established diagnostic criteria of ACR/EULAR (2010) as described [2]. Fifteen HV matched for age, sex and BMI were also recruited for the control group. Demographic, epidemiologic and treatment data of HV and RA patients are summarized in Table 1. The study protocol was approved by the local institutional review boards of CHU Hospital of Liège (Research Ethics Committee-human protocol #2005-020-Principal Investigator: Prof M. Malaise).

**Table 1:** Clinical characteristics of patients enrolled in the study for the explorative phase

	HV	RA
n	15	15
Female	15	15
Age—mean (*range*)	41 years (24–48)	43 years (25–49)
BMI—mean (*range*)	22 (19–28)	23 (17–36)
Disease duration—mean (*range*)	–	86 months (4–300)
DAS28-CRP	–	4.9 (3.6–8)
ESR	–	28.5 (2–67)
CRP+ %	–	61
RF+ %	–	66
Anti-CCP+ %	–	83
Treatment %		
Corticoids	–	40
NSAID	–	46
MTX	–	33
Leflunomide	–	13
Biologics	–	40

*HV* healthy volunteers, *RA* rheumatoid arthritis, *BMI* body mass index, *ESR* erythrocyte sedimentation rate, *CRP* C-Reactive Protein, *RF* rheumatoid factor; *anti-CCP* anti-cyclic citrullinated peptide, *NSAID* nonsteroidal anti-inflammatory drugs, *MTX* methotrexate

#### Treatment response

In order to evaluate the treatment response, 90 patients suffering from ERA, of the CAP48 cohort, were included in the study and blood samples were collected at time 0 (T0) and after 12 months of treatment (T12). The CAP48 cohort included ERA patients younger than 50 years old, with a disease duration < 3 months and naïve to DMARDs therapy. Ninety HV paired for age and sex were included as control subjects. The study was approved by Ethics Committee of the Cliniques Universitaires Saint-Luc (Bruxelles; Study No. B403201317717). Table 2 summarizes the data of participants which were included in the validation phase of the study.

**Table 2:** Clinical characteristics of patients enrolled in the study at T0 and T12

	HV	ERA	
n	90	90	
Female	72	72	
Age—mean (*range*)	34.4 (19–51)	34.1 (19–50)	
RF+ %	–	66	
Anti-CCP+ %	–	68	

Treatment %		T0	T12
Corticoids	–	–	11
MTX	–	–	93
Biologics	–	–	24
Hydroxychloroquine	–	–	5

Clinical measures		Mean (*range*)_T0	Mean (*range*)_T12
DAS28-CRP	–	4.4 (7.3–1.21)	2.7 (5.3–1.2)
SDAI	–	23.3 (70.4–0)	9.5 (77–0.1)
CDAI	–	21 (65.2–0)	8.2 (30–0)
TJC	–	9 (33–0)	2.9 (19–0)
TJC28	–	6.7 (26–0)	2.3 (19–0)
SJC	–	6.3 (27–0)	1.3 (8–0)
SJC28	–	4.8 (26–0)	1.1 (7–0)
CRP mg/dL	–	2.3 (26–0.09)	0.6 (10–0.02)
HAQ	–	1 (2.4–0)	0.7 (2.8–0)
VAS *med*	–	41.1 (89–10)	15.9 (70–0)
VAS *pat*	–	53.9 (100–0)	32.2 (90–0)
VAS *pain*	–	55 (100–0)	31 (90–0)
VAS *fatigue*	–	54.9 (100–0)	41.1 (96–0)

*HV* healthy volunteers, *ERA* early rheumatoid arthritis, *DAS28-CRP* disease activity score 28 joints, *SDA*: Simplified Disease Activity Index, *CDAI* Clinical Disease Activity Index, *TJC28* 28-Tender Joint Count, *SJC28* 28-Swollen Joint Count; *HAQ* Health Assessment Questionnaire, *VAS* Visual Analogue Scale

### Samples collection

Human blood samples were collected in standard conditions and allowed to coagulate in plain glass tubes. Serum was obtained after centrifugation at 2800 rpm for 10 min, room temperature. Supernatants were aliquoted and stored at − 80 °C until use.

### Chemicals

The Glycoprotein Isolation kit WGA and Concanavalin A (ConA), and ECL chemiluminescent reagents were purchased from Thermo Fisher Scientific (Waltham, MA, USA). Peptide-*N*-glycosidase F (PNGase F) and neuraminidase were from New England Biolabs (Ipswich, MA, USA) and from Merck (Darmstadt, Germany), respectively.

Enzyme-linked immunosorbent assay (ELISA) kits for C-Reactive Protein (CRP) and lipopolysaccharide-binding protein (LBP) were from R&D Systems (Minneapolis, MN, USA). ELISA kits for Serum Amyloid A (SAA) and Corticosteroid Binding Globulin (CBG), were purchased from Thermo Fisher Scientific and BioVendor (Brno, Czech Republic), respectively. Antibodies anti-CBG and ant-LBP were from Abcam (Cambridge, UK); antibody ant-Rabbit was from Cell Signaling Technology (Boston, MA, USA).

### Glycoprotein enrichment

Glycoproteins were isolated from 25 μL of serum using the lectin WGA and ConA immobilized on agarose. In detail, for the explorative phase, 3 pools of serum provided from 5 patients for each pool were prepared. Twenty-five microlitre of each pool were used for WGA and other 25 μL were used for ConA. For the validation phase, 90 sera from ERA at T0 and 90 sera from the same ERA patients at time T12, were individually processed with WGA. Isolation was carried out according to the manufacturer’s instructions of the kit and glycoproteins were eluted in 400 μL. Eluate and flow-through were immediately aliquoted and stored at − 80 °C until further use.

### Glycoproteomics

For glycoproteomics, we prepared 3 pools for each class of analysis (RA and HV), and each pool included 5 samples. Eluates of each pool obtained after glycoprotein enrichment (with WGA or ConA, separately) were analysed by a proteomic approach. The total protein content of each pool was determined using RC DC kit according manufacturers recommendations (Biorad, Hercules, CA). Fifteen microgram of each pool were adjusted to 30 µL volume with 50 mM ammonium bicarbonate solution. The samples were reduced by adding dithiothreitol (DTT) at 10 mM and incubated for 40 min at 56 °C under agitation; they were then alkylated using iodoacetamide (IAM) at 20 mM final concentration for 30 min at room temperature and protected from the light. A second reduction was carried out for 5 min at room temperature by adjusting the DTT concentration to 21 mM final concentration. The proteins were precipitated using the 2D clean-up kit (GE Healthcare, Chicago, IL) following manufacturers recommendations. The proteins pellets were resuspended in 50 mM ammonium bicarbonate and digested overnight using a 1/50 w/w trypsin (Pierce Proteomics Grade, Thermo Scientific)/proteins ratio at 37 °C under stirring. A second digestion step was carried out using a 1/100 (w/w) trypsin/proteins ratio and 80% acetonitrile (v/v) at 37 °C during 3 h under stirring. Digestion was stopped by adding trifluoroacetic acid (TFA) at 0.5% final (v/v). The samples were then dried under vacuum using a speedvac (Thermo Scientific, Bremen, Germany). The peptides were resuspended in water acidified with 0.1% (v/v) TFA. An aliquot corresponding to 3.5 µg was purified using C18 tips (Pierce, Thermo Scientific) and then dried. The sample were resuspended in 100 mM ammonium formate pH 10 at 2.5 µg per 9 µL and internal standard Mass Prep Digestion Standard (Waters, Milford, MA) was added in each sample at an amount of 150 fmol ADH per 9 µL.

2.5 µg of proteins (9 µL) were injected onto the 2D-nanoAcquity UPLC system (Waters, Milford, MA) hyphenated to a Q Exactive quadrupole orbitrap mass spectrometer (Thermo Scientific, Bremen, Germany) operated in positive nanoelectrospray. The configuration of the 2D-nanoUPLC system was a reversed phase pH 10 and reversed phase pH 3-based two dimension separation. The first dimension separation was made on an X-Bridge BEH C18 5 μm column (300 μm × 50 mm). The trap column Symmetry C18 5 μm (180 μm × 20 mm) and analytical column HSS T3 1.7 μm (75 μm × 250 mm) (Waters, Milford, CA) were used after an online dilution to lower pH values. The samples were loaded at 2 μL/min (20 mM ammonium formate solution adjusted to pH 10) on the first column and subjected to three isocratic elution steps (13.3%, 19% and 65% acetonitrile). Each eluted fraction was desalted on the trap column after a ten times online dilution to pH 3 and subsequently separated on the analytical column; flow rate 250 nL/min, solvent A (0.1% formic acid in water) and solvent B (0.1% formic acid in acetonitrile) with a linear gradient: 0 min, 99% A; 5 min, 93% A; 140 min, 65% A. The total run time for each of the fractions obtained by isocratic elution was 180 min. The LC eluent was directly electrosprayed from the analytical column at 2.1 kV voltage through the liquid junction of the nanospray source. The chromatography system was coupled to a Thermo Scientific Q Exactive Hybrid Quadrupole-orbitrap mass spectrometer (Thermo Scientific, Bremen, Germany), programmed for data-dependent acquisition mode. The mass spectrometer method was a Top12 MS/MS method, meaning that the spectrometer acquired one full MS spectrum, selected the 12 most intense peaks in this spectrum (singly charged precursors excluded) and made a full MS2 spectrum of each of these 12 compounds. The parameters for MS spectrum acquisition were: mass range from 400 to 1750 m/z, resolution of 70,000 (full width at half maximum), AGC target of 1 × 106 or maximum injection time of 200 ms. The parameters for MS/MS spectrum acquisition were: isolation window of 2.0 m/z, ion trap higher energy collision dissociation (HCD) fragmentation normalized collision energy (NCE) of 25, resolution of 17,500, AGC target of 1 × 105 or maximum injection time of 50 ms, dynamic exclusion: 10 s.

Proteins were identified using MaxQuant v.1.5.5.1 and quantified using Label-Free Quantification determination [31]. The main parameters were: database Uniprot restricted to human taxonomy (20,199 sequences, downloaded in January 2015), PSM and protein FDR of 1%, carbamidomethylation of cysteines as fixed modifications, Oxidation of Methionine and Acetylation of protein N-Term as variable modifications, minimum 2 peptides per protein including at least one unique peptide, match between run with a matching window of 0.7 min and an alignment window of 20 min.

### ELISA

The levels of CRP, SAA, LBP and CBG were detected by commercial ELISA kits according to the manufacturer’s instructions. The calibration ranges were 15.6–1000 pg/mL, 9.4–600 ng/mL, 0.78–50 ng/mL and 3.13–200 ng/mL for CRP, SAA, LBP and CBG, respectively. Serum was diluted 1:50,000, 1:1000, 1:1500, 1:500 for CRP, SAA, LBP and CBG respectively. The dilution for the eluate, after glycoprotein isolation with WGA and ConA, was 1:50. All experiments were performed in triplicate.

### Deglycosylation

Eluate and flow-through, obtained from WGA enrichment, were deglycosylated by PNGase F. First, samples were denatured during 10 min at 100 °C with Glycoprotein Denaturing Buffer (0.5% SDS, 40 mM DTT). Then, sodium phosphate (50 mM, pH 7.5), 1% NP-40 and PNGase F (450 U) were added to digest proteins for 1 h at 37 °C. The reaction was stopped by freezing. The treatment of eluates with neuraminidase required incubation (3 h, 37 °C) of samples with 2 U of enzyme at pH 5.5, according to the manufacturer’s instructions. The reaction was stopped by heating for 5 min at 100 °C. Products of reaction were visualized by western blot analysis.

### Western blot

CBG and LBP expression in eluate and flow-through, with or without PNGase F or neuraminidase treatment, was assessed by western blot analysis. Briefly, samples were run on 12% (for CBG) or 10% (for LBP and neuraminidase treatment) SDS-PAGE gels and transferred onto PVDF membranes. The membranes were then incubated with anti-CBG polyclonal antibody (0.3 μg/mL) or anti-LBP monoclonal antibody (1:2000 dilution). HRP-conjugated anti-Rabbit (1:1000 dilution) was used as a secondary antibody. Immunoblots were developed using the ECL chemiluminescent detection system.

### Statistical analysis

In the glycoproteomics analysis, label free quantitative intensities obtained for each glycoproteins identified by mass spectrometry were transformed as log2 values for normalization. Proteins were quantified using Label-Free Quantification determination as described by Cox et al. [31]. The accurate quantification has been obtained without isotopic labels with the MaxQuant software protein. In particular, with the MaxLFQ algorithms, it was possible to achieve the most accurate quantification by extracting the maximum ratio information from peptide signals. This quantification can be combined with the classical statistical tests. Statistical significance between [RA/HV]_WGA_ vs [RA/HV]_ConA_ for each glycoprotein normalized intensity was performed by the Student’s t-test. All data are presented as mean ± SD. In order to avoid that expression levels of glycosylated CBG and LBP after WGA enrichment was only due to initial expression levels of these two proteins in serum samples, concentrations measured in the WGA eluates were normalized with their corresponding total concentration obtained in serum by ELISA assay. Comparison between T0 and T12 of CBG and LBP levels in serum was performed using the paired Wilcoxon test for non-normal data; comparisons among healthy controls and ERA (T0 or T12) were performed using unpaired Kolmogorov–Smirnov test for non-normal data. Statistical significance between groups for normalized quantity of CBG and LBP in eluate was calculated by paired (for T0 vs T12 groups) or unpaired (for HV vs T0/T12) Student’s t-test. A P-value ≤ 0.05 was considered significant. Different statistical tests were performed according to comparison type (between paired or unpaired samples group). Paired tests were used for the comparison T0 vs T12 because same patients were compared before and after treatment. Unpaired tests were applied when ERA patients (T0 or T12) were compared with HV, evaluating different subjects. Moreover, Student’s t-test (paired or unpaired) was employed for parametric data. Wilcoxon (a paired test) and Kolmogorov–Smirnov (an unpaired test) were applied for non-parametric data.

To determine the statistical correlations among proteins and the clinical measures, Spearman’s rank correlation coefficient was calculated. A P-value ≤ 0.05 was considered significant. Statistical analysis was performed with Graph Pad Prism 6 software and SPSS software (SPSS/PC Statistical Package for the Social Science, update for 10.1., SPSS, Chicago, IL, USA, 2000).

## Results

### Experimental plan

In this study, we hypothesized that removal of terminal sugar residue (i.e. sialic acid) can occur for some glycoproteins, as already observed for IgG immunoglobulins. Accordingly, glycoproteins were enriched using two different lectins, WGA and ConA, described for their high affinity for sialic acid and mannose residues, respectively [32–34]. ConA was only used for normalization process as it was expected to remain constant in both groups.

Truncated glycoproteins without terminal sialic acid residue were not retained on lectin WGA in the RA group compared to healthy controls, whereas same glycoproteins were retained on ConA lectin via their mannose residues in both groups. The following ratio for each glycoprotein level was calculated indicating the highest rate of desialylation: [(RA/HV)_WGA_/(RA/HV)_ConA_]^−1^ if the ratio was > 1 and the highest rate of sialylation if the ratio was < 1.

This glycoproteomics study was divided in two phases (i) the explorative phase and (ii) the validation phase (Fig. 1). The explorative phase was dedicated to the identification by LC–MS/MS of desialylated glycoproteins isolated by lectin WGA from serum samples of RA patients (n = 15) compared to healthy controls (n = 15). After statistical analysis of proteomic data, two glycoproteins, CBG and LBP were selected as potential candidates presenting altered glycosylated residues in RA. They were confirmed by ELISA for each individual samples [RA (n = 15); HV (n = 15)] as potential candidates for the validation phase. In the validation phase, we have investigated the desialylation of CBG and LBP in ERA sera (n = 90) compared to controls (n = 90) and the status of glycosylation of both proteins after 1 year of treatments (n = 90).

### Exploratory phase

#### Glycoproteomics

**Fig. 2:**
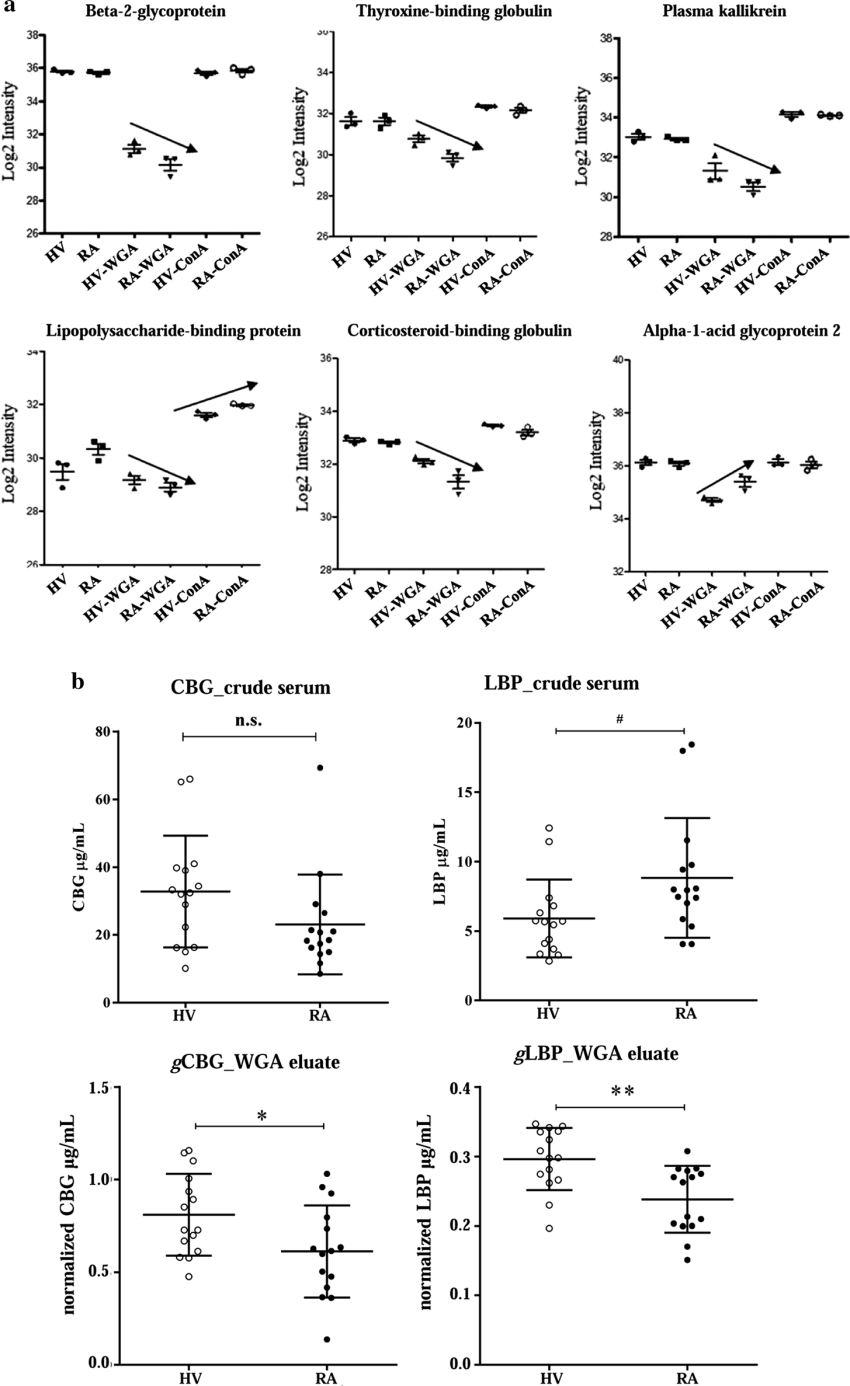
Results from the explorative phase. **a** Some glycoproteins which were found differentially expressed between HV and RA patients by LC/MS–MS analysis. **b** ELISA assay for CBG and LBP on serum and WGA eluate of HV and RA patients which were included in the explorative phase. Scatter dot plots represent (M) ± standard deviation (SD) of concentration (serum) or of normalized value (eluate); ^#^P-value ≤ 0.05 (Kolmogorov–Smirnov test); *P-value ≤ 0.05, **P-value ≤ 0.01 (unpaired t-test). *gCBG* glycosylated CBG, *gLBP* glycosylated LBP

Serum samples of RA patients (n = 15) and healthy controls (n = 15) were merged in 3 pools of 5 patients for each group, and loaded on lectin (WGA or ConA, separately) resins for glycoproteins enrichment. Glycoproteins eluates were then analysed by 2D-UPLC-Orbitrap MS/MS, which identified 247 glycoproteins whose only 191 proteins were detected in all pools. Sixteen glycoproteins (e.g. LBP and CBG) were selected according to (i) their highest desialylated rate [(WGA/ConA)^−1^ ratio > 1] and (ii) their statistical significance when comparing [RA/HV]_WGA_ ratio *vs* [RA/HV]_ConA_ ratio (P-values < 0.05) (Table 3). Three other proteins (i.e. adiponectin, alpha-1-acid glycoprotein 2 and cartilage acidic protein 1) were selected according to (i) their highest rate of sialylation as the (WGA/ConA)^−1^ ratio was < 1 and (ii) their statistical significance when comparing [RA/HV]_WGA_ ratio *vs* [RA/HV]_ConA_ ratio (P-values < 0.05) (Table 3). Some glycoproteins are represented in Fig. 2a. CBG and LBP glycoproteins were selected according to their role described in RA [25–29]. Their level of expression and glycosylated status were confirmed by ELISA for each sample before and after enrichment by lectins (Fig. 2b). For CBG, glycosylated protein levels were significantly decreased in RA compared to the healthy control group (P-value = 0.03) after WGA enrichment, whereas total levels of CBG in crude serum were not significant between RA and the healthy control group. For LBP, glycosylated protein levels were significantly decreased in RA compared to the control group (P-value = 0.002) after WGA enrichment, whereas total levels of LBP in crude serum were statistically increased between RA and control group (P-value = 0.03).

**Table 3:** Proteins found differentially expressed between HV and RA patients with LC/MS–MS analysis

Protein IDs	Protein names	Gene names	(WGA/ConA)^−1^	P-value
P02749	Beta-2-glycoprotein 1	APOH	2.18	2.69e^−05^
P05543	Thyroxine-binding globulin	SERPINA7	1.66	5.00e^−05^
P03952	Plasma kallikrein	KLKB1	1.65	3.36e^−03^
P18428	Lipopolysaccharide-binding protein	LBP	1.57	3.36e^−05^
O00533	Neural cell adhesion molecule L1-like protein	CHL1	1.52	3.77e^−03^
P00751	Complement factor B	CFB	1.47	3.43e^−03^
P36955	Pigment epithelium-derived factor	SERPINF1	1.47	2.83e^−05^
P08185	Corticosteroid-binding globulin	SERPINA6	1.44	1.44e^−03^
Q9UGM5	Fetuin-B	FETUB	1.40	4.42e^−03^
P02747	Complement C1q subcomponent subunit C	C1QC	1.35	2.34e^−03^
Q15485	Ficolin-2	FCN2	1.33	4.59e^−04^
P10643	Complement component C7	C7	1.29	6.10e^−03^
P08603	Complement factor H	CFH	1.20	8.52e^−03^
O75882	Attractin	ATRN	1.19	7.03e^−03^
P05154	Plasma serine protease inhibitor	SERPINA5	1.17	5.19e^−03^
P01031	Complement C5	C5	1.09	1.17e^−03^
Q15848	Adiponectin	ADIPOQ	0.83	4.20e^−03^
P19652	Alpha-1-acid glycoprotein 2	ORM2	0.57	2.02e^−05^
Q9NQ79	Cartilage acidic protein 1	CRTAC1	0.63	5.43e^−04^

#### Glycoprotein enrichment

**Fig. 3:**
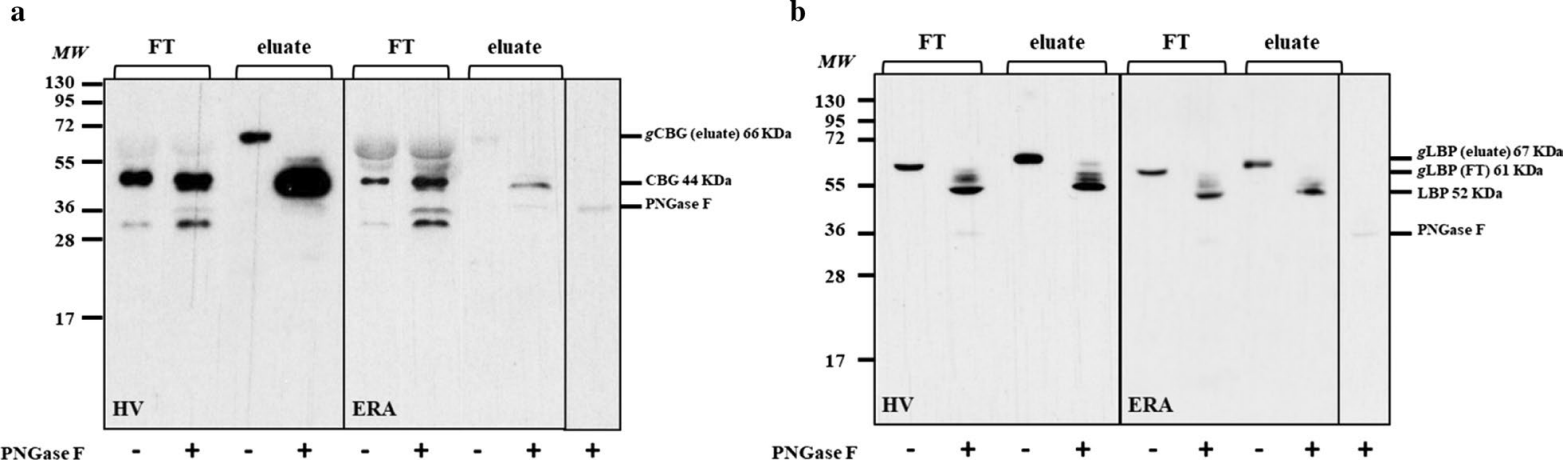
Western blot analysis for CBG and LBP. WB analysis for CBG (**a**) and LBP (**b**) in serum from healthy volunteer (HV) and from early rheumatoid arthritis patient (ERA). *FT* flow-through, *gCBG* glycosylated CBG, *gLBP* glycosylated LBP, *MW* molecular weight markers

To evaluate the efficiency of glycoproteins enrichment, a western blot was performed on the eluate and flow-through for both CBG and LBP glycoproteins (Fig. 3a, b). As expected, glycosylated CBG (66 kDa) was enriched in the elution fraction and was not present in the flow-through (Fig. 3a). When treated with PNGase F, the eluate showed that CBG molecular weight (MW) decreased from 66 to 44 kDa, while no change was found in the flow-through (Fig. 3a). For LBP, we detected in the eluate and flow-through two distinct bands with different MW, 67 and 61 kDa respectively (Fig. 3b). Moreover, after deglycosylation, we observed for both fractions the LBP at 52 kDa, which is the theoretical MW for LBP (Fig. 3b).

This experiment illustrates that WGA lectins were not saturated by glycoproteins of interest in ERA and healthy control conditions. For CBG, PNGase F digestion experiment indicates that the eluate contains the glycosylated form of the protein whereas the flow-through contains the non-glycosylated form. For LBP, two different glycosylated forms are present, one is retained by the WGA lectin whereas the other one is not, but both are digested by PNGase F.

#### Desialylation

**Fig. 4:**
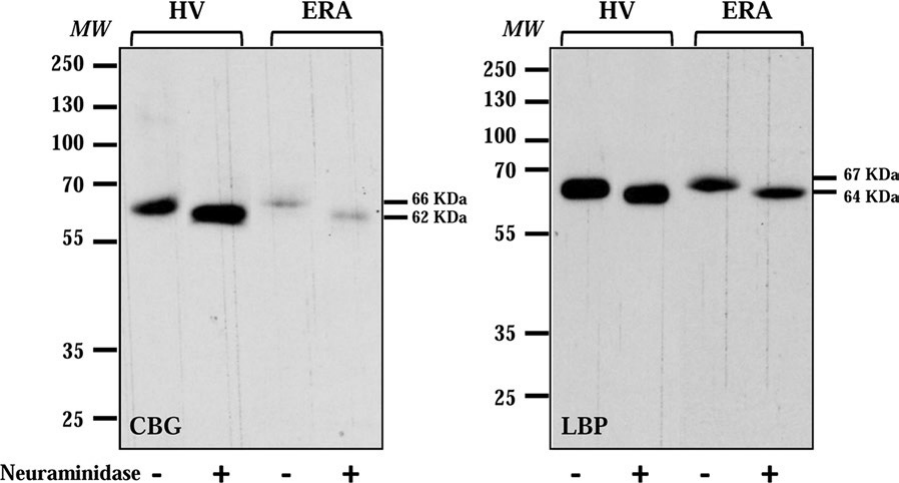
Desialylation. WB analysis for CBG and LBP of eluates in the presence or not of neuraminidase. *HV* healthy volunteer, *ERA* early rheumatoid arthritis, *MW* molecular weight markers

In order to validate that CBG and LBP linked to WGA contained sialic acid, we performed neuraminidase treatment. We used neuraminidase from Arthrobacter ureafaciens which cleaves sialic acids residues by hydrolysis of α(2 → 6), α(2 → 3), α(2 → 8), and α(2 → 9) linkages. After desialylation we observed that CBG and LBP molecular weight shifted from 66 to 62 kDa and from 67 to 64 kDa, respectively, which demonstrated that glycoproteins retained by WGA actually contained terminal sialic acid residues (Fig. 4).

### Validation phase and treatment response

#### Markers of inflammation

**Fig. 5:**
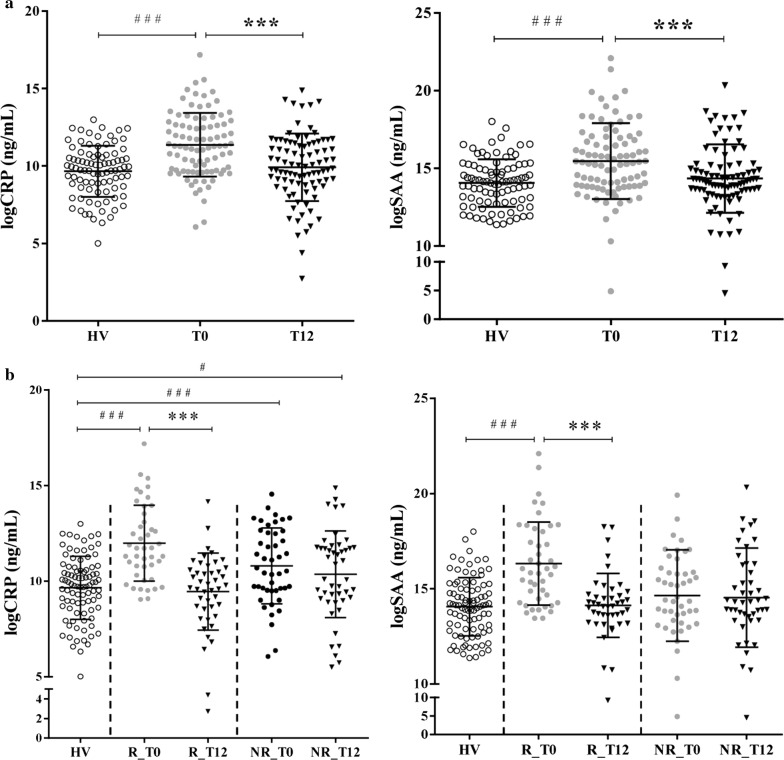
ELISA assay for CRP and SAA. **a** ELISA assay for CRP and SAA on serum from healthy volunteers (HV) and ERA patients at time T0 and after 12 months of treatment (T12). The log transformation was applied to concentration values of CRP and SAA. Scatter dot plots represent the M ± SD. ^###^P-value ≤ 0.001 (HV vs RA; unpaired t-test); ***P-value ≤ 0.001 (T0 vs T12; paired t-test). **b** Variation of CRP and SAA in good (R) and non (NR) responders. ^#^P-value ≤ 0.05; ^###^P-value ≤ 0.001 (HV vs RA; unpaired t-test); ***P-value ≤ 0.001 (T0 vs T12; paired t-test)

Before the validation phase, we quantified two acute-phase proteins, CRP and serum amyloid A proteins in sera of the validation cohort. Both proteins levels increased in patients population at time T0 compared to healthy controls (P-value ≤ 0.001); moreover, after 12 months of treatment, their values decreased significantly (both P-value ≤ 0.001), while that between HV and T12 were not significant (Fig. 5a).

Then, the patients were divided in good (R, n = 44) and non (NR, n = 46) responders according to the EULAR good response observed at T12 (Additional file 1), defined as an improvement > 1.2 in the DAS28-CRP from baseline (T0), and a DAS attained during follow-up (T12) < 3.2 [35–37]. All patients that filled these criteria were included in the R group, all the others in the NR group. Patients of our cohort showed a mean of DAS28-CRP of 4.6 at T0 for the R group which decreased to 1.9 at T12, while the mean was 4.2 in the NR group, decreasing to 3.5 after treatment. In the R group there were 82% of patients in remission (DAS28-CRP ≤ 2.6) at T12 and 19% in the NR group.

When we examined the variation in R and NR groups, CRP and SAA levels decreased significantly in the group of R between T0 and T12 (P-value ≤ 0.001) but not in the NR group (Fig. 5b). Moreover we observed a positive correlation between DAS28-CRP and CRP (Spearman’s rank correlation coefficient 0.514; P-value = 2.98e^−07^), and SAA (Spearman’s rank correlation coefficient 0.474; P-value = 3.05e^−06^) (Table 4). It is noteworthy to observe that higher levels of CRP and SAA at T0 are related to better response to treatment. Indeed, in the R group respect to NR group, we can observe a higher correlation between CRP (T0) and ΔCRP (T0–T12), the same observation has done for SAA (Additional file 2). In addition, there are significantly higher levels of CRP and SAA in T0 for the R group respect to NR group, with a P-value of 0.006 and 0.0009, respectively (R_T0 vs NR_T0, unpaired t-test).

**Table 4:** Clinical correlations in the R and NR group (T0 and T12)

	DAS28-CRP	SDAI	CDAI	TJC	TJC28	SJC	SJC28	HAQ	VAS med	VAS pat	VAS pain	VAS fatigue
R group												
SAA												
a	3.05e^−06^	1.04e^−05^	5.14e^−05^	2.6e^−04^	4.22e^−04^	2.53e^−06^	6.0e^−05^	0.01	1.54e^−05^	0.002	1.7e^−04^	0.003
b	0.474	0.451	0.418	0.382	0.368	0.480	0.414	0.274	0.445	0.321	0.393	0.323
CRP												
a	2.98e^−07^	1.52e^−06^	1.83e^−05^	4.35e^−05^	6.82e^−05^	2.18e^−06^	5.58e^−05^	0.022	3.05e^−06^	4.3e^−04^	7.9e^−03^	0.009
b	0.514	0.487	0.440	0.424	0.411	0.483	0.416	0.243	0.477	0.368	0.459	0.282
CBG_serum												
a	n.s	n.s	n.s	n.s	n.s	n.s	n.s	n.s	n.s	n.s	n.s	n.s
b												
CBG_eluate												
a	0.005	0.014	n.s	0.037	0.016	n.s	n.s	n.s	0.048	n.s	n.s	n.s
b	− 0.298	− 0.262		− 0.224	− 0.256				− 0.213			
LBP_serum												
a	6.34e^−06^	3.21e^−05^	1.23e^−04^	3.59e^−04^	3.49e^−04^	5.19e^−06^	4.33e^−05^	0.004	1.0e^−04^	0.002	4.4e^−04^	0.025
b	0.461	0.428	0.398	0.374	0.373	0.467	0.421	0.306	0.405	0.326	0.369	0.244
LBP_eluate												
a	0.004	0.014	0.037	0.009	0.003	n.s	n.s.	n.s	0.047	n.s	n.s	n.s
b	− 0.303	− 0.261	− 0.222	− 0.280	− 0.309				− 0.214			
NR group												
SAA												
a	n.s	n.s	n.s	n.s	n.s	n.s	n.s	n.s	n.s	n.s	n.s	0.009
b												0.284
CRP												
a	n.s	n.s	n.s	n.s	n.s	n.s	n.s	n.s	0.048	n.s	n.s	n.s
b									0.208			
CBG_serum												
a	n.s	n.s	n.s	n.s	n.s	n.s	n.s	n.s	n.s	n.s	n.s	n.s
b												
CBG_eluate												
a	n.s	n.s	n.s	n.s	n.s	n.s	n.s	n.s	n.s	n.s	n.s	n.s
b												
LBP_serum												
a	0.005	0.048	n.s	n.s	n.s	0.036	n.s	n.s	0.04	n.s	n.s	n.s
b	0.292	0.206				0.22			0.216			
LBP_eluate												
a	n.s	n.s	n.s	n.s	n.s	n.s	n.s	n.s	n.s	n.s	n.s	n.s
b												

To determine the statistical correlations among proteins and the clinical measures, the Spearman’s rank correlation coefficient was calculated. Only the significant correlations are indicated; a: P-value; b: correlation coefficient

*n.s* not significant

#### Validation of the glycoproteins CBG and LBP levels in response to treatment

**Fig. 6:**
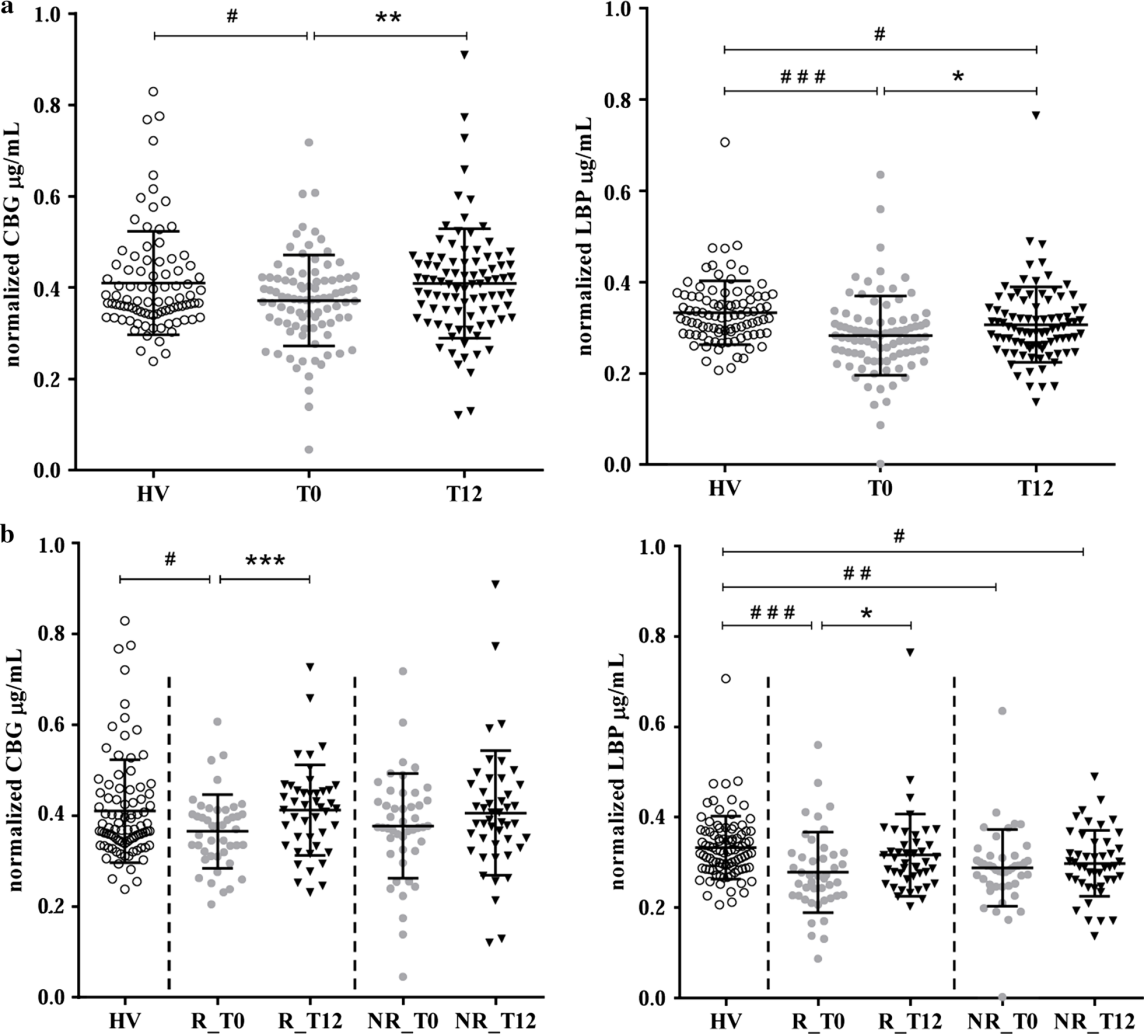
Validation for CBG and LBP with ELISA. **a** ELISA assay for CBG and LBP on WGA eluates after glycoproteins enrichment in HV and ERA patients at time T0 and T12. **b** Their variation in good (R) and non (NR) responders. Scatter dot plots represent M ± SD of the normalized values. ^#^P-value ≤ 0.05; ^##^P-value ≤ 0.01; ^###^P-value ≤ 0.001 (HV vs ERA; unpaired t-test); *P-value ≤ 0.05, **P-value ≤ 0.01 (T0 vs T12; paired t-test)

The validation of CBG and LBP levels after glycoprotein enrichment by WGA was widened on a larger cohort of ERA patients (n = 90) and healthy controls (n = 90), but also analysing the response to treatment after 12 months. Accordingly, CBG and LBP were dosed on serum before glycoprotein enrichment (Additional file 3) and on the glycosylated fraction (Fig. 6a) after WGA isolation. We confirmed the decrease of both proteins, in their glycosylated form, in patients at time T0 compared with HV (for CBG: P-value = 0.017; for LBP: P-value ≤ 0.0001). While, after treatment, values raised significantly compared with values at T0 (for CBG: P-value = 0.006; LBP P-value = 0.04). Furthermore, the glycosylated CBG and LBP showed a negative correlation with DAS28-CRP. The Spearman’s rank correlation coefficients (r) were significant for CBG (r = − 0.298; P-value = 0.005) and for LBP (r = − 0.303; P-value = 0.004), compared with DAS28-CRP, in the R group (Table 4). Indeed, in the R group, the difference between T0 and T12 was significant (for CBG: P-value = 0.0006; for LBP: P-value = 0.04) while it was not in NR group (Fig. 6b).

#### Clinical correlations

Besides correlations indicated above, all the significant correlations (P-value ≤ 0.05) found among proteins and clinical measures in R and NR groups are summarized in Table 4. Correlations calculated for all patients are summarized in table of Additional file 4. CBG levels in serum did not correlate with any clinical measure while CBG in the eluate showed a negative correlation with DAS28-CRP, SDAI, TJC, TJC28 and VAS. Concerning LBP, its expression in serum was positively correlated with all the clinical parameters, while the correlation became negative among LBP in the eluate and DAS28-CRP, SDAI, CDAI, TJC, TJC28 and VAS.

## Discussion

In the present work, we applied LC–MS/MS and immunoassays to monitor glycosylation changes during the course and treatment of RA. Indeed, glycan-modifications can underpin biological effects that initiate or alter the course of the disease. At present, studies were mainly focused on Ig [17]. Evidence suggests that the activity and the course of RA strongly correlate with the increase proportion of agalactosylated (IgG-G0) structures [38], and treatment also seems to revert the agalactosylation process [6]. Glycosylation analysis is challenging regarding the plethora of glycan structures, also influenced by many factors [17, 39, 40]. For example, age and sex have a substantial effect on serum N-glycosylation [17, 40, 41], and individual changes of IgG glycosylation are described in inflammatory response [18]. Therefore, the positive outcome of a glycoproteomic study is largely related to the quality of a well-matched patients cohort, as observed in our study. Another critical point is the glycosylation enrichment by lectins regarding to the risk of saturation, which can affect the differential rate between protein levels and which was controlled by western blotting in our study. While the glycosylated form of CBG was detected only in the eluate, glycosylated LBP has been found both in the eluate and flow-through, but with diverse molecular weight. Actually, two different glycosylation forms for LBP were described [42], and WGA cannot recognise all glycans. WGA is a specific lectin with a strong affinity for sialic acid and in a less extent for *N*-acetyl glucosamine-containing glycans. Actually, by desialylation of the eluates, we observed that CBG and LBP isolated by WGA contained terminal sialic acids residues. Indeed, it has been estimated that the affinity of WGA for sialic acid is four times greater than that for *N*-acetyl glucosamine [32, 43]. ConA is a well described lectin with a strong affinity for mannose residues [33, 34, 44–46]. Both lectins were used in the explorative phase: (i) WGA for investigating the desialylation rate of glycans and (ii) ConA for its constant binding to mannose in the control and RA group. The rate of desialylated proteins after WGA enrichment in the RA group compared to the controls was calculated and normalized to the same ratio obtained after ConA enrichment. Nineteen glycoproteins were highlighted as potentially modified for their glycosylation status in RA condition. Some of them were already described as glycoproteins [26, 47] such as thyroxine-binding globulin or beta-2-glycoprotein [1, 48, 49] some others as being involved in RA pathology [29, 30, 50] (e.g. alpha-1-acid glycoprotein 2 whose fucosylation and sialylation have shown to significantly increase in RA) [51] or other chronic inflammatory diseases [52–54].

Two proteins, CBG and LBP were selected and confirmed by ELISA in the explorative phase, then validated on a larger cohort investigating treatment response.

Good and non-responders were defined according to the EULAR good response observed at T12 [35–37]. So far, it remains unknown why some patients better respond than others. DMARD, especially methotrexate, is currently the most applied therapy in the early treatment phase of ERA, even though response can differ among patients. Generally, patients with more active RA better respond to treatment [55], as actually observed in our cohort. However, RA is a polygenic disease whose treatment response depends on many factors such as age, sex, psychological factors, disease activity and duration [56, 57]. Four major phenotypes of RA synovium have been highlighted: lymphoid, myeloid, low inflammatory and fibroid, each presenting various molecular and cellular heterogeneities that can impact clinical outcome to therapies [58]. To date, treatment is based on clinician’s experience but a better knowledge of the relationship between pathogenic molecular drivers in RA and therapeutic response could guide to use more rational and effective treatment for different patients.

In the present study, inflammation level was also quantified by measuring the expression level of two acute-phase reactant proteins: CRP and SAA highly expressed in chronic inflammatory diseases, including RA [59–62]. In our analysis, ERA patients exhibited raised levels of CRP and SAA compared with HV. After 12 months of treatment, the same expression was significantly decreased. Besides, CRP and SAA levels were significantly correlated with clinical parameters. Further, when patients were divided in good and non-responders, according to the variation of DAS28-CRP between T0 and T12, the decrease of CRP and SAA levels after treatment was not anymore significant in the group of non-responders, on the contrary to the good responders group.

Regarding to CBG and LBP glycoproteins, our study pointed out for the first time a modification of their glycosylation status according to ERA activity.

RA is extensively recognized as a steroid-responsive disease, and CBG is the major protein for binding, transport and release of anti-inflammatory corticosteroids. CBG is a highly glycosylated protein, with 6 known sites of N-glycosylation, for which *N*-glycans can modulate its function and structure [26, 63]. However, there is still little consensus on the role played by the glycosylation of CBG. It has been proposed that both delivery and binding of cortisol can be affected. Studies showed that the delivery of cortisol to inflamed tissues is related to the cleavage of CBG which can be reduced by altered glycosylation in RA. This can interfere with cortisol delivery contributing to perpetrate inflammation [25, 64]. On the other hand, *N*-glycans are necessary for steroid binding [26] and it has been suggested that glycosylation state could decrease during inflammation, affecting binding affinity of CBG [64, 65]. In line with this, we observed a significant reduction of CBG glycosylation in ERA patients compared with controls. This firstly emerged with LC–MS/MS analysis, and was subsequently confirmed by ELISA not only with the subjects included in the LC–MS/MS analysis, but also with the 90 patients and 90 controls enrolled for the validation phase. Further, we examined the effect of a 12 months treatment and observed that glycosylated CBG levels increased significantly, which confirmed that the altered glycosylation status of CBG was linked to RA activity. Moreover, there was also a significant negative correlation with clinical parameters such as DAS28-CRP and SDAI (Table 4, Additional file 4). It is noteworthy to observe the marked and significant difference in glycosylated CBG level between non and good responders. Hence, these data contribute to sustain that, while total CBG levels are not affected in RA, glycosylation changes of CBG are strictly related to the disease activity score and respond to treatment. We observed an alteration in glycosylation also for LBP.

LBP is an acute-phase protein which binds LPS, a glycolipid present on the membrane of Gram-negative bacteria. Therefore, LBP appears to be directly involved in the recognition of pathogenic bacteria, delivers endotoxin and enhances LPS-mediated cytokine induction [66, 67]. Indeed, the complex between LPS and LBP binds to CD14 receptor promoting the production of cytokines through the activation of TLR4 signalling [68, 69]. LBP is associated with autoimmune diseases and local infectious diseases [29, 70–72], and its levels are higher in RA [28–30]. In line with these studies, we pointed out a significant increase of total LBP levels in serum from ERA patients regarding to healthy controls (Additional file 3) which was reversed in the same ERA patients after 12 months of treatment. Indeed, LBP concentration decreased again to values comparable to those of healthy subjects. In addition, LBP detected in serum was positively correlated with clinical parameters and there was a significant trend towards increasing total LBP with increasing RA disease activity as measured by DAS28-CRP (Additional file 5). This is in agreement with previous studies which proposed LBP as a sensitive serum biomarker to evaluate RA course in active RA patients, considering that LBP was significantly correlated with RA disease activity parameters [28–30]. Concerning its post translational modification, LBP hold four known sites of N-glycosylation whose function remains unexplained [66, 73]. We observed in ERA a significant decrease of glycosylated LBP level compared to controls, which was, as observed for CBG, reverted after 12 months-period of treatment. Moreover, glycosylated LBP was negatively correlated with DAS28-CRP (Table 4, Additional file 4). This affected the difference between good and non-responders.

## Conclusions

The present work succeeded in pointing out glycosylation changes in RA. Until now, they have been mainly characterized for IgG, whereas we focused on two new glycoproteins. Indeed, we noticed that RA modified the glycosylation status of CBG, which is related to RA and its clinical features, while total CBG was not affected. LBP is already a marker for the disease, but in addition we detected that also the decrease of its glycosylation can be related to RA. Our work suggests that the observed glycosylation changes are predominantly quantitative. Nevertheless, the structural characterization of CBG and LBP *N*-glycans is required and should be performed by other glycoproteomic techniques to obtain glycosylation site-specific information. To define glycosylation structures and sites is surely a technical complex task, but advances in MS-based methods should provide useful tools. This could overtake the limitation which we acknowledge to our study. Indeed, we proposed an association among RA and the reduction of glycosylation for CBG and LBP, but the exact molecular background for these changes cannot be answered by this observational work. However, the strength is the comprehensive laboratory and clinical evaluation which enables to observe that glycosylation change of CBG and LBP is undoubtedly strictly related to RA and its course. Moreover, these data demonstrated that these alterations in CBG and LBP glycosylation respond to treatment.

In conclusion, our overarching results provide insight into how differences in CBG and LBP glycosylation are related to RA course and treatment. Overall, the present study can pave the way for future functional studies for a better knowledge about the consequence of this post translational modification in RA.

## Supplementary information

**Additional file 1.** Variation of DAS in patients. Variation of DAS28-CRP at time T0 and after 12 months of treatment (T12) between good (R) and non (NR) responders. A value of DAS28-CRP ≤ 2.6 indicates remission.

**Additional file 2.** ELISA assay for CRP and SAA. **a**) Relation between CRP(T0) and ΔCRP(T0–T12) **b**) Relation between SAA(T0) and ΔSAA(T0–T12), in good and non responders. r: Spearman’s rank correlation coefficient.

**Additional file 3.** ELISA assay for CBG and LBP on serum. ELISA assay for CBG (**a**) and LBP (**b**) on serum of HV and ERA patients at time T0 and T12. Scatter dot plots represent M ±  SD of concentration; ^#^P-value ≤ 0.05, ^###^P-value ≤ 0.001 (HV vs ERA; Kolmogorov–Smirnov test); ***P-value ≤ 0.01 (T0 vs T12; Wilcoxon matched-pairs signed rank test).

**Additional file 4.** Table of clinical correlations for all patients (T0 and T12). To determine the statistical correlations among proteins and the clinical measures, the Spearman’s rank correlation coefficient was calculated. Only the significant correlations are indicated; a: P-value; b: correlation coefficient; n.s.: not significant.

**Additional file 5.** ELISA assay for LBP on serum. ELISA assay for LBP on serum of ERA patients, depicted according to RA disease activity. DAS stands for DAS28-CRP. Scatter dot plots represent M ± SD of concentration; ^#^P-value ≤ 0.05; ^##^P-value ≤ 0.01; ^###^P-value ≤ 0.001 (Kolmogorov–Smirnov test). DAS28-CRP ≤ 2.6 remission; 2.6 < DAS28-CRP ≤ 3.2 low activity; 3.2 < DAS28-CRP ≤ 5.1 moderate activity; DAS28-CRP > 5.1 high activity.

## Data Availability

The datasets used and analysed during the current study are available from the corresponding author on reasonable request.

## Abbreviations

CBG: corticosteroid-binding globulin
CDAI: Clinical Disease Activity Index
ConA: concanavalin A
CRP: C-Reactive Protein
DAS: disease activity score
DMARDs: disease modifying antirheumatic drugs
DTT: dithiothreitol
ERA: early rheumatoid arthritis
HV: healthy volunteers
IAM: iodoacetamide
LBP: lipopolysaccharide-binding protein
LC–MS/MS: liquid chromatography–mass spectrometry/mass spectrometry
LPS: lipopolysaccharide
MW: molecular weight
NR: non responder
PNGaseF: peptide-*N*-glycosidase F
R: responder
RA: rheumatoid arthritis
SAA: serum amyloid A
SDAI: Simplified Disease Activity Index
SDS: sodium dodecyl sulfate
T0/T12: time 0/12
TFA: trifluoroacetic acid
TJC: Tender Joint Count
VAS: Visual Analogue Scale
WGA: wheat germ agglutinin
