# Supplementary material for: Glycosylation deficiency of lipopolysaccharide-binding protein and corticosteroid-binding globulin associated with activity and response to treatment for rheumatoid arthritis
Source: J Transl Med. 2020 Jan 6;18:8. doi: 10.1186/s12967-019-02188-9 (PMC6945416; doi:10.1186/s12967-019-02188-9)
Supplement: Supplementary file 1 — Additional file 1. Variation of DAS in patients. Variation of DAS28-CRP at time T0 and after 12 months of treatment (T12) between good (R) and non (NR) responders. A value of DAS28-CRP ≤ 2.6 indicates remission. [file 12967_2019_2188_MOESM1_ESM.pdf]

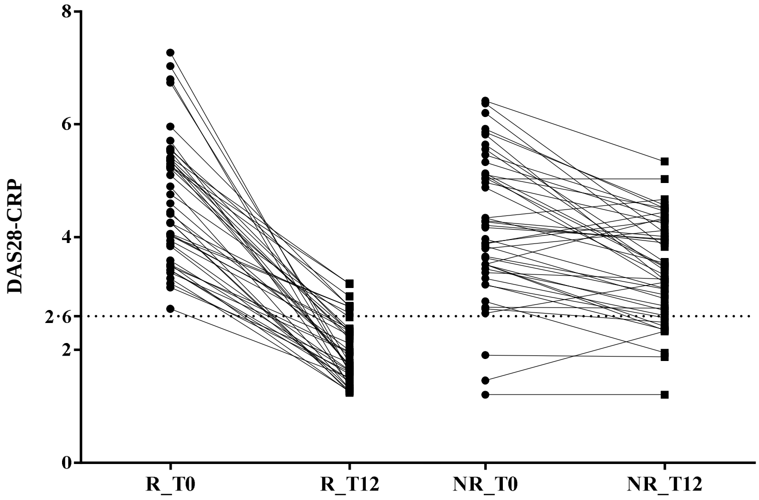

Additional file 1. Variation of DAS28-CRP at time T0 and after 12 months of treatment (T12) between good (R) and non (NR) responders. A value of DAS28-CRP ≤ 2.6 indicates remission.
