# Supplementary material for: Glycosylation deficiency of lipopolysaccharide-binding protein and corticosteroid-binding globulin associated with activity and response to treatment for rheumatoid arthritis
Source: J Transl Med. 2020 Jan 6;18:8. doi: 10.1186/s12967-019-02188-9 (PMC6945416; doi:10.1186/s12967-019-02188-9)
Supplement: Supplementary file 2 — Additional file 2. ELISA assay for CRP and SAA. a) Relation between CRP(T0) and ΔCRP(T0–T12) b) Relation between SAA(T0) and ΔSAA(T0–T12), in good and non responders. r: Spearman’s rank correlation coefficient. [file 12967_2019_2188_MOESM2_ESM.pdf]

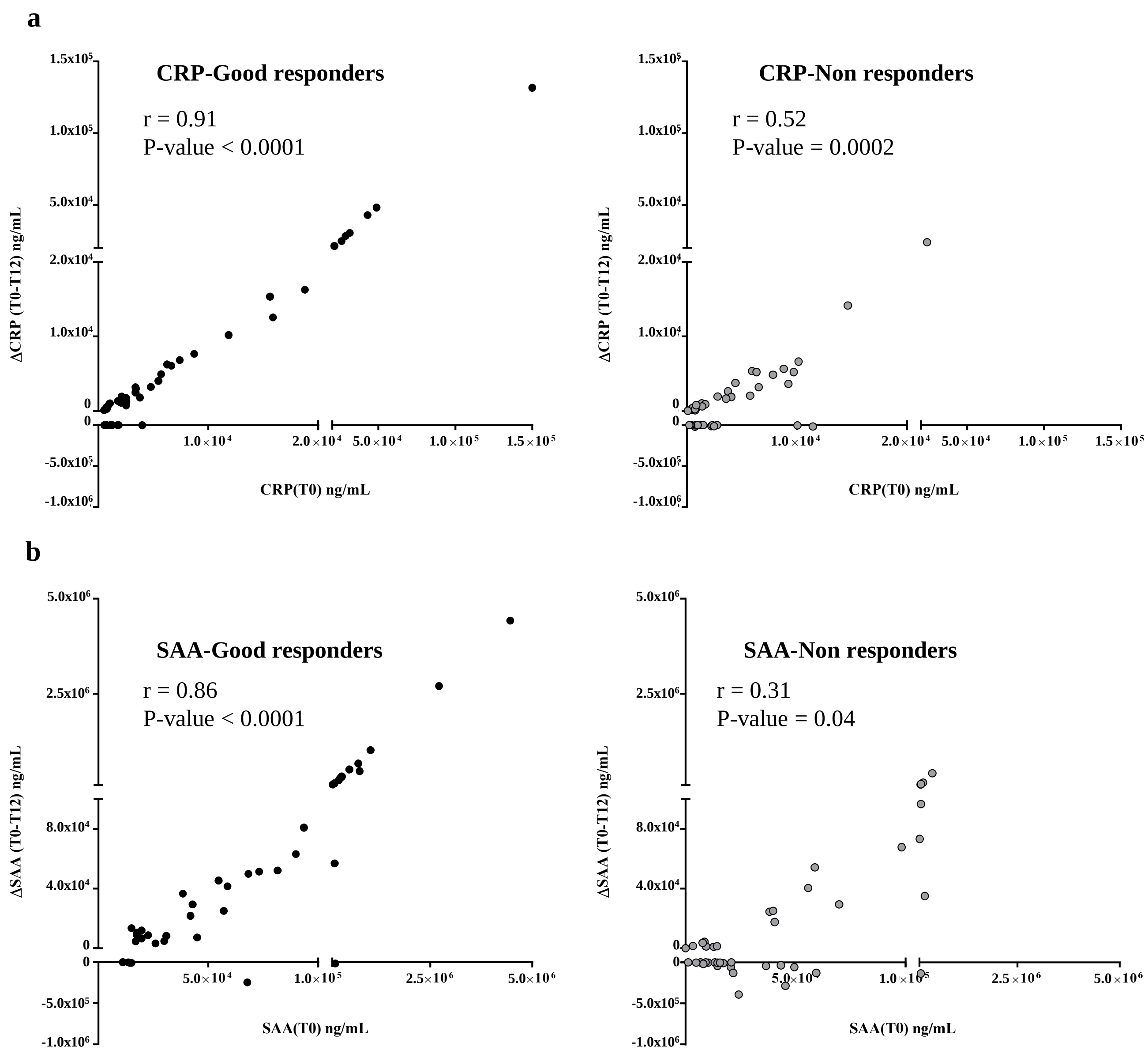

Additional file 2. a) Relation between CRP(T0) and ΔCRP(T0-T12) **b)** Relation between SAA(T0) and ΔSAA(T0-T12), in good and non responders. r: Spearman's rank correlation coefficient.
