# Supplementary material for: Glycosylation deficiency of lipopolysaccharide-binding protein and corticosteroid-binding globulin associated with activity and response to treatment for rheumatoid arthritis
Source: J Transl Med. 2020 Jan 6;18:8. doi: 10.1186/s12967-019-02188-9 (PMC6945416; doi:10.1186/s12967-019-02188-9)
Supplement: Supplementary file 3 — Additional file 3. ELISA assay for CBG and LBP on serum. ELISA assay for CBG (a) and LBP (b) on serum of HV and ERA patients at time T0 and T12. Scatter dot plots represent M ± SD of concentration; #P-value ≤ 0.05, ###P-value ≤ 0.001 (HV vs ERA; Kolmogorov–Smirnov test); ***P-value ≤ 0.01 (T0 vs T12; Wilcoxon matched-pairs signed rank test). [file 12967_2019_2188_MOESM3_ESM.pdf]

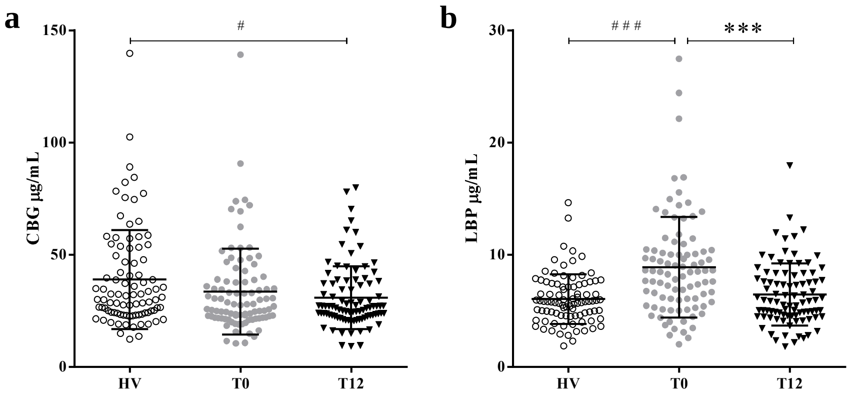


**Additional file 3.** ELISA assay for CBG (**a**) and LBP (**b**) on serum of HV and ERA patients at time T0 and T12. Scatter dot plots represent M ± SD of concentration; # p-value ≤ 0.05, # # # p-value ≤ 0.001 (HV vs ERA; Kolmogorov-Smirnov test); *** p-value ≤ 0.01 (T0 vs T12; Wilcoxon matched-pairs signed rank test).
