# Supplementary material for: Glycosylation deficiency of lipopolysaccharide-binding protein and corticosteroid-binding globulin associated with activity and response to treatment for rheumatoid arthritis
Source: J Transl Med. 2020 Jan 6;18:8. doi: 10.1186/s12967-019-02188-9 (PMC6945416; doi:10.1186/s12967-019-02188-9)
Supplement: Supplementary file 4 — Additional file 4. Table of clinical correlations for all patients (T0 and T12). To determine the statistical correlations among proteins and the clinical measures, the Spearman’s rank correlation coefficient was calculated. Only the significant correlations are indicated; a: P-value; b: correlation coefficient; n.s.: not significant. [file 12967_2019_2188_MOESM4_ESM.docx]

**Additional file 4.** Clinical correlations for all patients (T0 and T12).

|  |  | **DAS28-CRP** | **SDAI** | **CDAI** | **TJC** | **TJC 28** | **SJC** | **SJC 28** | **HAQ** | **VAS med** | **VAS**  **pat** | **VAS pain** | **VAS fatigue** |
| --- | --- | --- | --- | --- | --- | --- | --- | --- | --- | --- | --- | --- | --- |
| **SAA** | ***a*** | 0·001 | 0·007 | 0·029 | n.s. | n.s. | 0·0002 | 0·006 | n.s. | 0·001 | n.s. | 0·023 | n.s. |
|  | ***b*** | *0·238* | *0·201* | *0·163* |  |  | *0·280* | *0·203* |  | *0·246* |  | *0·172* |  |
| **CRP** | ***a*** | 1·05e^-07^ | 2·2e^-05^ | 0·0002 | 0·003 | 0·004 | 9·31e^-06^ | 0·001 | 0·022 | 5·11e^-07^ | 0·002 | 2·3e^-05^ | n.s. |
|  | ***b*** | *0·384* | *0·311* | *0·277* | *0·225* | *0·215* | *0·325* | *0·245* | *0·171* | *0·366* | *0·229* | *0·314* |  |
| **CBG_serum** | ***a*** | n.s. | n.s. | n.s. | n.s. | n.s. | n.s. | n.s. | n.s. | n.s. | n.s. | n.s. | n.s. |
|  | ***b*** |  |  |  |  |  |  |  |  |  |  |  |  |
| **CBG_eluate** | ***a*** | 0·002 | 0·012 | 0·039 | n.s. | n.s. | n.s. | n.s. | n.s. | 0·005 | n.s. | 0·038 | n.s. |
|  | ***b*** | *-0·226* | *-0·188* | *-0·154* |  |  |  |  |  | *-0·208* |  | *-0·157* |  |
| **LBP_serum** | ***a*** | 6·93e^-07^ | 1·86e^-05^ | 0·0001 | 0·002 | 0·008 | 1·58e^-06^ | 0·0001 | 0·008 | 2·61e^-05^ | 0·0018 | 0·001 | n.s. |
|  | ***b*** | *0·360* | *0·313* | *0·286* | *0·226* | *0·197* | *0·351* | *0·284* | *0·196* | *0·310* | *0·231* | *0·251* |  |
| **LBP_eluate** | ***a*** | 0·012 | 0·036 | n.s. | 0·038 | n.s. | 0·042 | n.s. | n.s. | n.s. | n.s. | n.s. | n.s. |
|  | ***b*** | *-0*·*188* | *-0·157* |  | *-0·155* |  | *-0·153* |  |  |  |  |  |  |

To determine the statistical correlations among proteins and the clinical measures, the Spearman’s rank correlation coefficient was calculated. Only the significant correlations are indicated; a: p-value; b: correlation coefficient; n.s.: not significant.
