# Supplementary material for: Glycosylation deficiency of lipopolysaccharide-binding protein and corticosteroid-binding globulin associated with activity and response to treatment for rheumatoid arthritis
Source: J Transl Med. 2020 Jan 6;18:8. doi: 10.1186/s12967-019-02188-9 (PMC6945416; doi:10.1186/s12967-019-02188-9)
Supplement: Supplementary file 5 — Additional file 5. ELISA assay for LBP on serum. ELISA assay for LBP on serum of ERA patients, depicted according to RA disease activity. DAS stands for DAS28-CRP. Scatter dot plots represent M ± SD of concentration; #P-value ≤ 0.05; ##P-value ≤ 0.01; ###P-value ≤ 0.001 (Kolmogorov–Smirnov test). DAS28-CRP ≤ 2.6 remission; 2.6 < DAS28-CRP ≤ 3.2 low activity; 3.2 < DAS28-CRP ≤ 5.1 moderate activity; DAS28-CRP > 5.1 high activity. [file 12967_2019_2188_MOESM5_ESM.pdf]

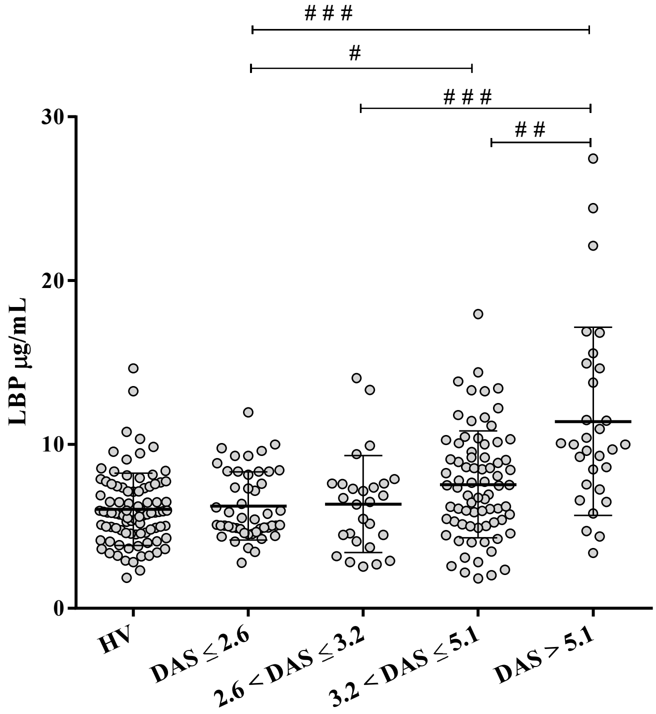

Additional file 5. ELISA assay for LBP on serum of ERA patients, depicted according to RA disease activity. DAS stands for DAS28–CRP. Scatter dot plots represent M ± SD of concentration; # p-value ≤ 0.05; ## p-value ≤ 0.01; # # # p-value ≤ 0.001 (Kolmogorov-Smirnov test). DAS28–CRP ≤ 2.6 remission; 2.6 < DAS28–CRP ≤ 3.2 low activity; 3.2 < DAS28–CRP ≤ 5.1 moderate activity; DAS28–CRP > 5.1 high activity.
